# Synthesis of 3-(Imidazo[2,1-*b*]thiazol-6-yl)-2H-chromen-2-one Derivatives and Study of Their Antiviral Activity against Parvovirus B19

**DOI:** 10.3390/molecules24061037

**Published:** 2019-03-15

**Authors:** Ilaria Conti, Rita Morigi, Alessandra Locatelli, Mirella Rambaldi, Gloria Bua, Giorgio Gallinella, Alberto Leoni

**Affiliations:** 1 Department of Pharmacy and Biotechnology, Alma Mater Studiorum-University of Bologna, Via Massarenti 9, 40138 Bologna, Italy; ilaria.conti5@unibo.it (I.C.); gloria.bua2@unibo.it (G.B.); giorgio.gallinella@unibo.it (G.G.); 2 Department of Pharmacy and Biotechnology, Alma Mater Studiorum-University of Bologna, Via Belmeloro 6, 40126 Bologna, Italy; rita.morigi@unibo.it (R.M.); mirella.rambaldi@unibo.it (M.R.); alberto.leoni@unibo.it (A.L.); 3 Unit of Microbiology, Alma Mater Studiorum-University of Bologna, S. Orsola-Malpighi Hospital, Via Massarenti 9, 40138 Bologna, Italy

**Keywords:** parvovirus B19, coumarins, antiviral activity, imidazo[2,1-*b*]thiazole

## Abstract

Parvovirus B19 (B19V) is a human pathogenic virus associated with a wide range of clinical conditions. Currently, there are no recognized antiviral drugs for B19V treatment; therefore, efforts in the search for compounds inhibiting B19V replication are now being pursued. Coumarins (chromen-2-ones) are considered a privileged structure for designing novel orally bioavailable and non-peptidic antiviral agents. To further contribute to the development of new drugs against B19V, our research was focused on the synthesis, characterization and evaluation of antiviral activity of some new 3-(imidazo[2,1-*b*]thiazol-6-yl)-2H-chromen-2-one derivatives. The effects of the synthesized compounds on cell viability and viral replication were investigated by employing two relevant cellular systems, the myeloblastoid cell line UT7/EpoS1 and primary erythroid progenitor cells (EPCs). Some of the tested compounds showed inhibitory activity both on cell viability and on viral replication, depending on the cellular system. These results suggest that the mechanism involved in biological activity is sensitive to small structural changes and that it is possible to direct the activity of the 3-(imidazo[2,1-*b*]thiazol-6-yl)-2H-chromen-2-one core.

## 1. Introduction

Coumarins (chromen-2-ones) are already in use as therapeutic agents in human. They are considered a privileged structure for designing novel orally bioavailable and non-peptidic antiviral agents endowed with a characteristic pharmacophore: A planar hydrophobic aromatic nucleus connected with a lactone group which is both responsible for hydrogen bond formation and a facilitator of protein-ligand binding [1]. Many natural products and synthetic compounds having coumarin nucleus have been evaluated for inhibitory effects against HIV replication, and some of them have been found to inhibit different stages in the HIV replication cycle [2,3,4,5,6]. Moreover, several coumarin analogues also proved to have antiviral activities against herpes simplex virus type 1 (HSV-1), herpes simplex virus type 2 (HSV-2), hepatitis virus [7,8,9], chikungunya virus (CHIKV) and influenza A virus [10].

Among the pathogenic human viruses still lacking a specific antiviral therapy, parvovirus B19 (B19V), an ssDNA virus in the family Parvoviridae, is one of the most relevant [11,12]. This virus is characterized by a selective but not exclusive tropism for erythroid progenitor cells (EPCs) in the bone marrow [13], and by a strict dependence on the cellular machinery and environment for its replication [14,15]. In the population, infection is widespread and can be associated with an ample range of pathologies and clinical manifestations, from the asymptomatic or mild to the severe and in some cases life-threatening [16]. The selective tropism for erythroid progenitor cells in the bone marrow can cause a partial block in erythropoiesis that may manifest as transient or persistent erythroid aplasia. Common systemic manifestations of infection are erythema infectiosum in children and post-infection arthropathies mainly affecting adults; however, the virus has been implicated in a growing collection of other different pathologies, among them myocarditis, encephalitis and connective tissue diseases. Notably, infection in pregnancy may be transmitted to the foetus, posing a risk of foetal death and/or foetal hydrops [17].

The incomplete characterization of viral proteins and of the molecular mechanisms involved in viral replication still hampers the rational design of specifically targeted drugs, while the demanding in vitro cell culture conditions of B19V limit the feasibility of setting up a high throughput screen against the available chemical libraries. So B19V suffers a gap in the development of antiviral strategies. A VLP-based vaccine is technically feasible but still under development [18], and until now no specific antiviral therapy has been clinically evaluated. Current therapeutic options are limited and mostly symptomatic or supportive, in particular intravenous immunoglobulins (IVIG) are considered the only available option to neutralise infectious virus and control infection in case of incompetence of the immune system [19]. However, beneficial effects of supportive treatments are temporally limited, and even IVIG are seldom able to clear infection unless a patient’s own antiviral immune response becomes effective. Therefore, continued efforts in the research and development of antiviral drugs possessing a specific inhibitory activity against B19V should be considered a relevant goal to increase the range and efficacy of available therapeutic or preventive interventions.

To overcome all these problems, our group previously took two alternative approaches, based on a drug repositioning strategy, and on investigating known antiviral compounds for a possible activity against B19V. The first approach yielded antiviral activity provided by the cell-proliferation inhibitor hydroxyurea (HU) [20]. The second approach yielded the acyclic nucleoside phosphonate cidofovir (CDV) [21,22], although with suboptimal activity, and thereafter its lipid conjugate brincidofovir (BCV) [23], which strikingly shows activity comparable to what was obtained against other DNA viruses and presents promising properties as a broad-range antiviral drug [24].

To further contribute to the development of new drugs against B19V, our research was focused on the synthesis of some new 3-(imidazo[2,1-*b*]thiazol-6-yl)-2H-chromen-2-one derivatives. This scaffold, variously substituted, has already been investigated for potential anticancer activity [25]. Considering our pluriannual experience in the synthesis of 3-(imidazo[2,1-*b*]thiazol-5-ylmethylene)indolin-2-one [26], we planned the preparation of hybrid compounds (**6**–**10**) featuring a 2-indolinone, an imidazo[2,1-*b*]thiazole and a coumarine. According to a consolidated synthetic strategy, these three cores were linked to afford an extended hydrophobic scaffold bearing a free NH group, a carbonyl moiety and a lactone group which, by increasing the polarity, can promote the solubility and improve the binding affinity by forming H-bonds. To better define the SAR, we also subjected the starting synthones 3-(imidazo[2,1-*b*]thiazol-6-yl)-2H-chromen-2-ones **1a**,**b** [27,28], and 6-(2-oxo-2H-chromen-3-yl)imidazo[2,1-*b*]thiazole-5-carbaldehydes **2a**,**b**, to biological assays.

The potential activity of these compounds in a selective inhibition of B19V replication was then assessed in two relevant cellular systems, the myeloblastoid cell line UT7/EpoS1 and primary erythroid progenitor cells (EPCs). Herein we report the results of this study.

## 2. Results

### 2.1. Chemistry

**Scheme 1:**
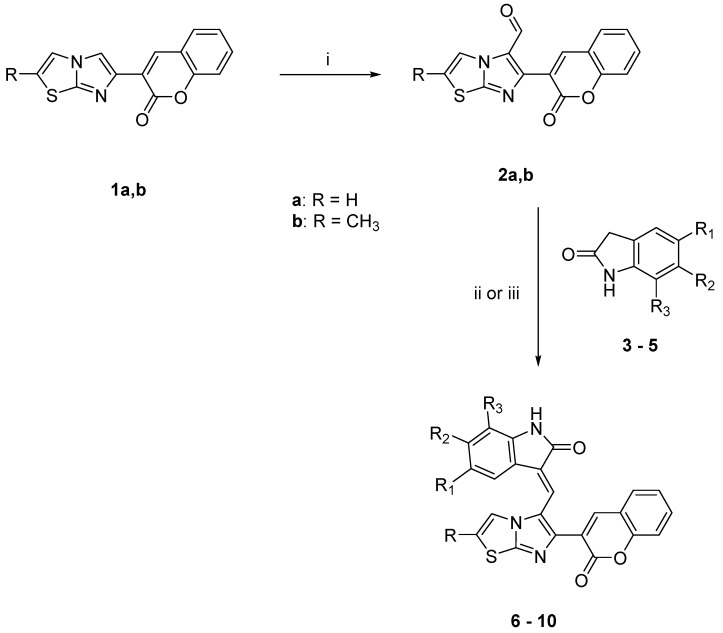
Synthetic route for obtaining compounds **6**–**10**. Reagents and conditions: (**i**) POCl_3_/DMF, CHCl_3_, 0 °C to rt, then reflux; (**ii**) CH_3_OH, piperidine; (**iii**) CH_3_OH, HCl.

Compounds **6**–**10** (see Scheme 1 and Table 1) were synthesized by means of the Knoevenagel reaction between the aldehydes **2a**,**b** and the appropriate oxindole. The reaction was performed in methanol in the presence of piperidine to obtain compounds **6**, **8**, **10**; and in presence of HCl 37% to obtain compounds **7** and **9**.

The new starting aldehydes **2a**,**b** were obtained by means of the Vilsmeier reaction on the 3-(imidazo[2,1-*b*]thiazol-6-yl)-2H-chromen-2-ones **1a**,**b**.

For R_1_, R_2_ and R_3_ see Table 1.

**Figure 1:**
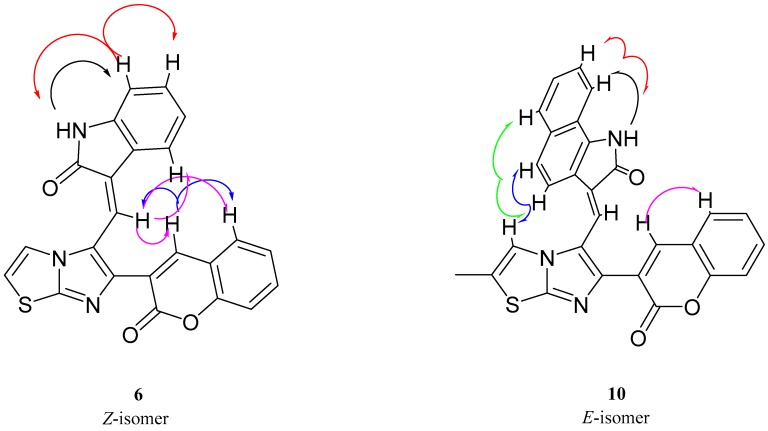
E and Z isomers of 3-(imidazo[2,1-*b*]thiazol-6-yl)-2H-chromen-2-one derivatives.

The IR, ^1^H NMR and ^13^C NMR spectra of the new compounds are in agreement with the assigned structures. The expected compounds were obtained as pure geometrical isomers. The geometrical configuration was determined by performing NOE experiments on derivatives **6** and **10** in order to evaluate whether the methine bridge and the proton at the 4 position of the indole (ind-4) are close in space (*Z* configuration) or not (*E* configuration) (Figure 1). First of all, we studied compound **6** and we noticed that the irradiation of the NH group (10.48 ppm) gave NOE at 6.87 ppm: this peak was therefore assigned to the hydrogen at the 7 position of the indole system (ind-7). The subsequent irradiation (ind-7) confirmed the spatial connection previously observed with the NH group and a second one with the triplet at 7.21 ppm, which therefore corresponds to the hydrogen at the 6 position of the indole system (ind-6). When the singlet corresponding to the proton at the 4 position of the coumarin moiety (cum-4, 8.40 ppm) was irradiated, NOE was observed at 7.96 ppm (methine bridge) and 7.86 ppm (cum-5). Afterward, irradiation of the methine bridge at 7.96 ppm gave NOE at 8.40 ppm (cum-4), 7.86 ppm (cum-5) and the indole proton at the 4 position (ind-4, 7.67 ppm). This last NOE experiment, by revealing the spatial closeness of the methine bridge and ind-4, confirms the *Z* configuration.

Analogous experiments on derivative **10** started with the irradiation of the NH group (11.39 ppm), which gave NOE at the doublet at 8.12 ppm: this peak was therefore assigned to the hydrogen at the 9 position of the benzoindole moiety (ind-9). The subsequent irradiation of ind-9 confirmed the spatial connection previously observed with the NH group and a second one with the multiplet at 7.48 ppm, which therefore corresponds to the hydrogen at the 8 position of the benzoindole moiety (ind-8). Further irradiation of the singlet corresponding to the proton at the 4 position of the coumarin moiety (cum-4, 8.53 ppm), gave NOE at 7.83 ppm (cum-5). Afterward, irradiation of the doublet at 6.64 ppm gave NOE at 7.25 ppm and at 7.45 ppm. Therefore, the doublet at 6.64 ppm was assigned to the hydrogen at the 4 position of the benzoindole moiety (ind-4), the doublet at 7.25 ppm was assigned to the hydrogen at the 5 position of the benzoindole moiety (ind-5, same J of ind-4) and the one at 7.45 ppm was assigned to the proton of the thiazole moiety. Finally, the irradiation of ind-5 gave NOE at 6.64 ppm (ind-4) and 7.78 ppm (ind-6). After these NOE experiments, we assigned the *E* configuration to compound **10**, and we believe that, at least in DMSO solution, it assumes the twisted configuration corresponding to the intermediate structure reported in Figure 1, because our studies did not reveal the spatial proximity of the methine bridge and ind-4, or a connection between the methine group and the proton at the 4 position of the coumarin (cum) system.

Finally, by means of a COSY experiment, we assigned all the other signals to the different protons.

The geometrical configuration of the other synthesized compounds was assigned by comparing the signals of ^1^H-NMR spectra. Derivatives **7–9** were found to be *Z* isomers since we noticed that in *Z* configuration protons at position 4 of the indole ring gave a doublet at about 7.30–7.60 ppm, whereas in *E* isomers this doublet is around 6.60 ppm.

### 2.2. Biological Studies

Two in vitro cellular systems that are established and appropriate for B19V were used for this study, primary erythroid progenitor cells (EPCs) and the UT7/EpoS1 cell line. In vitro-derived EPCs constitute a heterogeneous cellular population analogous to the primary target cells in the bone marrow and that present full permissiveness to viral replication depending on differentiation stage and proliferation rate. The human myeloblastoid cell line UT7/EpoS1 is a commonly used cell line that presents susceptibility to viral infection and a restricted pattern of permissiveness, allowing replication and transcription of the viral genome, along with a relatively limited yield of infectious virus production.

In our work, we first determined the maximal concentration of each compound that could be solubilised in cell culture conditions in the presence of 1% DMSO, previously determined as its highest concentration non-toxic to cells. Then, we determined the effects of these compounds at their maximal concentration, on cell viability and viral replication in both UT7/EpoS1 and EPCs cellular systems.

#### 2.2.1. Compound Solubility

For each compound, starting from stock solutions at 5 mM in DMSO, 1:2 serial dilutions were prepared within the range of 50 to 0.39 µM, in cell culture medium containing 1% DMSO (final volume of 100 µL, in 96 plate wells), and in parallel in medium with 1% DMSO added to both UT7/EpoS1 cells and EPCs (50,000 cells in 100 µL, in 96-well plate). After 48 h of incubation at 37 °C, the formation of precipitates was evaluated by visual inspection at optical microscopy. The maximal concentration of compounds that did not form precipitates in either condition is reported in Table 2, and was retained as the maximal concentration for use in subsequent experiments.

#### 2.2.2. Effects on Cell Viability

**Figure 2:**
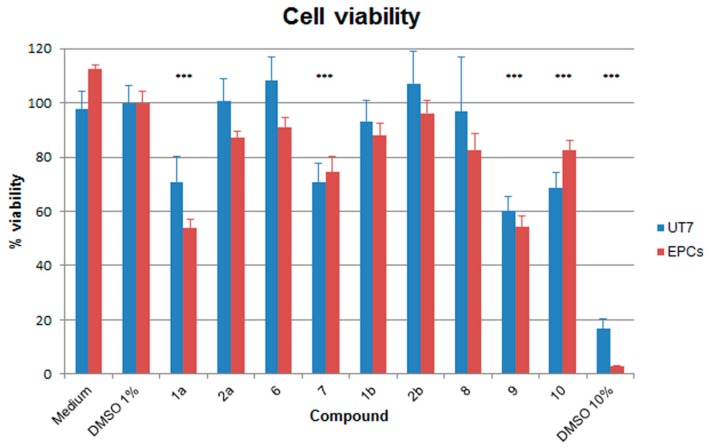
Percentage of viability of the UT7/EpoS1 and EPCs cells for different compounds compared to medium with 1% DMSO. The amounts of formazan dye produced by cells from the WST-1 reagent (CCK8 assay, Microtech) was measured as absorbance (OD) values, replicate net OD values were normalized with respect to the control samples and expressed as mean percentage values. Data were collected from triplicate wells in two different experiments; the statistical analysis was performed by one-way ANOVA (analysis of variance) followed by Dunnett’s multiple comparison test. *** *p* value <0.0001.

Effects of compounds on cell viability were determined in both UT7/EpoS1 and EPCs cell systems by a WST-8-based assay, a reagent that produces a formazan dye upon reduction in response to metabolic activity (CCK8 assay, Microtech). The cells were cultured for 48 h at 37 °C in medium containing 1% DMSO and each compound at its maximum concentrations of use established by the previous solubility tests. As controls, cells were also cultured in medium w/o DMSO, medium with 1% DMSO and medium with 10% DMSO. Results are reported in Figure 2, expressed as percentage viability with respect to medium with 1% DMSO. Reduction in cell viability was more pronounced in EPCs than UT7/EpoS1 cells, an effect already evident by culturing in 1% DMSO alone. However, presence of 1% DMSO did not alter cell viability to a significant extent in either cell system, while an almost complete loss of cell viability was induced by culturing in 10% DMSO. A relevant and significant reduction in cell viability, below 80% of control samples, was induced in both cellular systems by compounds 1a, 7, 9 and 10 (in this latter case, less for EPCs than UT7/EpoS1 cells).

#### 2.2.3. Antiviral Activity against B19V

Effects of compounds on B19V was evaluated in a 48 h course of infection. Both UT7/EpoS1 cells and EPCs were infected with B19V at the multiplicity of infection (moi) of 10^4^ geq/cell, then cultured in the presence of each compound at its maximal concentration of use. Infection was also conducted in medium w/o DMSO, and medium with 1% DMSO as controls. Cells were collected at 2 and 48 h post-infection (hpi), and the extent of viral replication was assessed by qPCR evaluation of the amount of viral genomes present in the cell system at 48 hpi, compared to the inoculum virus present at 2 hpi (Table 3).

**Figure 3:**
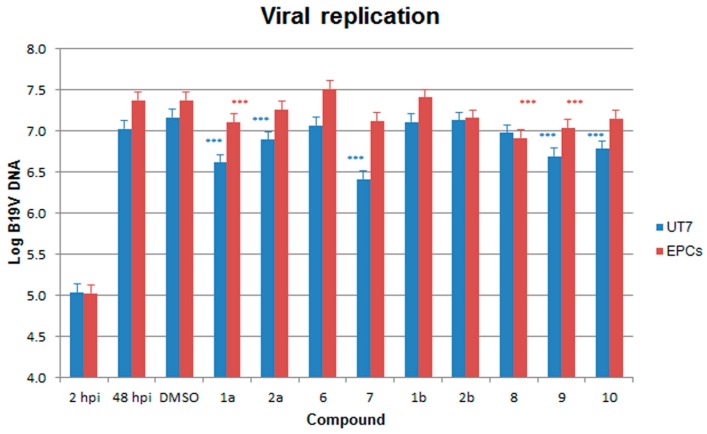
Amount of B19V DNA (Log geq/10^4^ cells) in the different samples, at 2 hpi and at 48 hpi following a course of infection in the presence of different compounds as indicated. Data were collected from duplicate experiments; the statistical analysis was performed by one-way ANOVA (analysis of variance) followed by Dunnett’s multiple comparison (control medium with 1% DMSO). *** *p* value < 0.0001.

**Figure 4:**
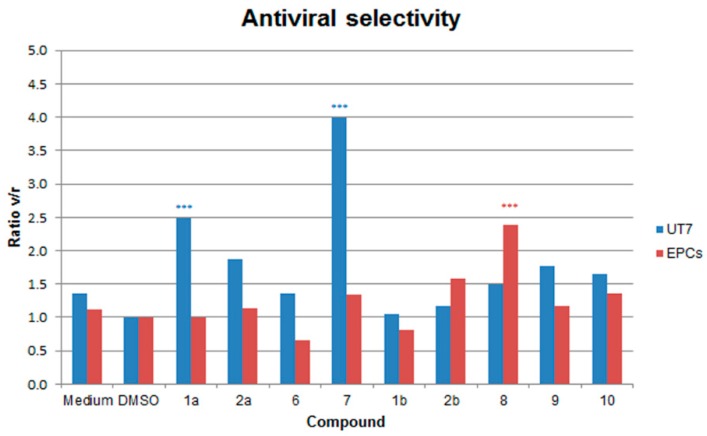
Selectivity of inhibition of B19V replication relative to cell viability (ratio v/r), each compared to medium with 1% DMSO. Data were collected from duplicate experiments; the statistical analysis was performed by one-way ANOVA (analysis of variance) followed by Dunnett’s multiple comparison test (control medium with 1% DMSO). *** *p* value < 0.0001.

**In UT7/EpoS1 cells.** When compared to the 2 hpi samples, a ~2 Log increase in the amount of viral DNA was evident at 48 hpi in control samples, from 5.03 to 7.02–7.16 Log geq/10^4^ cells. None of the compounds caused a complete inhibition of viral replication at 48 hpi, with final values in the range 6.41–7.13 Log geq/10^4^ cells (Figure 3). When compared to the control samples (1% DMSO), a significant reduction in the amount of B19V DNA at 48 hpi occurred in the presence of the compounds **1a**, **2a**, **7**, **9** and **10**. However, this reduction could be related to the effects on the cells and not on their direct action against the virus, then, as a measure of selectivity in the inhibition of viral replication, the ratio between residual cell viability and viral replication was evaluated (ratio v/r). A relevant (v/r > 2) and statistically significant selectivity was observed only for compounds **1a** and **7** (Figure 4).

**In EPCs.** When compared to the 2 hpi samples, a ~2 Log increase in the amount of viral DNA was also evident at 48 hpi in control samples (w/o DMSO, from 5.02 to 7.37 Log geq/10^4^ cells, in 1% DMSO, from 5.02 to 7.37 Log geq/10^4^ cells). Similarly, none of the compounds caused a complete inhibition of viral replication at 48 hpi, with final values in the range 6.91–7.51 Log geq/10^4^ cells (Figure 3). Effects of compounds were partly discordant with respect to UT7/EpoS1 cells. In particular, with respect to controls, a significant reduction in the amount of B19V DNA at 48 hpi occurred in the presence of the compounds **1a**, **8** and **9**, while compounds **6** and **1b** appeared to favour viral replication. By evaluating the ratio between residual cell viability and viral replication (ratio v/r), a relevant (v/r > 2) and statistically significant selectivity was observed only for compound **8**, while compound **6** confirmed its pro-viral effect (v/r < 0.6) (Figure 4).

**Figure 5:**
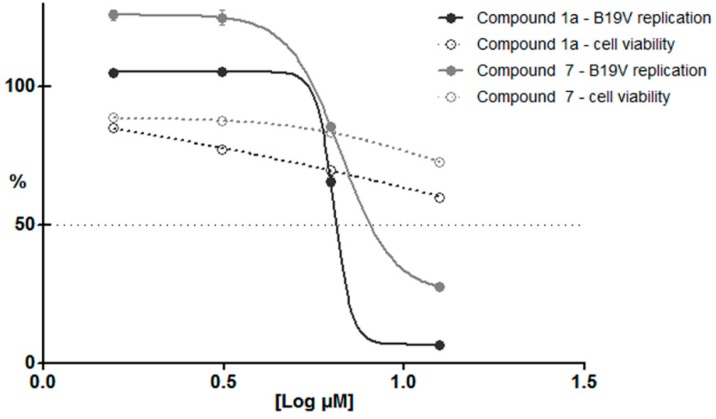
Viral replication and cell viability, normalized response to compounds **1a** and **7** in UT7/EpoS1 cells. Normalized dose-response curves were calculated from qPCR and CCK8 assay data, obtained at 48 hpi for the different experimental series and expressed as percentage values compared to control samples (DMSO 1%).

Compounds **1a** and **7** showed the highest activity; however, they were among those compounds that could be tested at the highest concentration of 12.5 µM. Thus, these were also tested in UT7/EpoS1 cells at lower concentrations, for both antiviral and cell toxicity activities. Results are shown in Figure 5. Concerning antiviral activity, a steep dose-response curve was obtained, where the two higher concentrations tested could inhibit viral replication compared to control samples (calculated EC_50_ values ~ 6.4–6.7 µM). On the other hand, cell viability was also affected, but with a different and less significant dose-response curve. This discrepancy suggests that these two effects were not directly related to each other, and that some degree of specific antiviral activity could be attributed to tested compounds over a background of a more generic cytotoxic effect.

## 3. Discussion

Although all compounds were used at their maximum concentrations, above which they form precipitates in cell culture conditions, none could completely inhibit viral replication. However, some of the compounds tested showed inhibitory activity both on cell viability and on viral replication, depending on the cellular system. Compounds **1a**, **7**, **9** and **10** caused a reduction in the viability of the UT7/EpoS1 cells, and for the compounds **1a** and **7**, a selective reduction in viral replication was induced. Compounds **1a**, **7** and **9** caused a reduction of cell viability also in EPCs, while only compound **8** induced a selective reduction in viral replication.

Based on the biological data, one can outline a scaffold profile that could be developed for subsequent studies, although the compounds tested are relatively few to define the relationships structures activities and did not show an important antiviral activity.

It is interesting to note that the 3-(imidazo[2,1-*b*]thiazol-6-yl)-2H-chromen-2-one core (**1a**) without the indolinone already shows a modest selective reduction in viral replication and a reduction of cell viability and that the simple substitution of the thiazole proton in position 2 with a methyl annuls these effects (**1b**).

Considering the derivatives (**6**–**10**) with the 3-((6-(2-oxo-2H-chromen-3-yl)imidazo[2,1-*b*]thiazol-5-yl)methylene)indolin-2-one scaffold, compounds **6** (*Z* –isomer) and **10** (*E*-isomer) do not display appreciable activity, whereas compounds **7** and **8**, both isomers *Z*, respectively show a weak selective reduction in viral replication in the UT7/EpoS1 cells (**7**) and in the EPCs (**8**). Based on these results it can be supposed that the spatial position of the indolinone fragment with respect to the imidazo[2,1-*b*]thiazole bicycle could affect the activity (**7**-*Z* and **8**-*Z* versus 10-*E*).

In particular, the compound **6** has the same backbone as **7** and **8** but does not bear the substituents which characterize them: a methoxy group in position 5 in the indolinone (**7**), a methyl group in position 2 in imidazo[2,1-*b*]thiazole (**8**); compound **9** has a hybrid structure: stackable both at **7** and at **8**, in effect it carries both substituents.

Overall, these results suggest that the mechanism involved in biological activity is sensitive to small structural changes (**7** and **8** versus **6** and **9**) and that it is possible to direct the activity of the pharmacophore 3-((6-(2-oxo-2H-chromen-3-yl)imidazo[2,1-*b*]thiazol-5-yl)methylene)indolin-2-one towards a cellular type: methoxy (**7**) for UT7/EpoS1 cells, methyl (**8)** for the EPCs cells.

The diverse effects observed in the different cellular environments may be related either to different uptake mechanisms and the resulting intracellular concentrations attained in the two cell types, or to different interference mechanisms towards replication machineries formed by a complex of the viral NS and cellular proteins, in this case suggesting a cellular protein as a more likely target.

## 4. Materials and Methods

### 4.1. Chemistry

All the compounds prepared have a purity of at least 98%, as determined by combustion analysis. The melting points are uncorrected. Reaction progress was monitored by TLC plates pre-coated with silica gel 60 F254 (Sigma Aldrich, Milan, Italy) visualized by UV (254 nm). Flash and gravity column chromatography were performed on Kieselgel 60 (Merck): the eluent was a mixture of petroleum ether/acetone in various proportions. The IR spectra were recorded in nujol on a Nicolet Avatar 320 E.S.P. (Thermo Fischer Scientific, Waltham, Massachusetts, USA); ν_max_ is expressed in cm^−1^. The ^1^H NMR and ^13^C NMR spectra were recorded on a Varian MR 400 MHz (ATB PFG probe) (Agilent, Palo Alto, CA, USA); the chemical shift (referenced to solvent signal) is expressed in δ (ppm). Multiplicities are quoted as s (singlet), d (doublet), t (triplet) and m (multiplet), br (broad) with coupling constants defined as *J* given in Hz (abbreviations: ar = aromatic, cum = coumarin, ind = indole, th = thiazole). Compounds were named relying on the naming algorithm developed by CambridgeSoft Corporation (Perkin Elmer, Milan, Italy) and used in Chem-BioDraw Ultra 14.0 (Perkin Elmer, Milan, Italy). All solvents and reagents, unless otherwise stated, were supplied by Aldrich Chemical Co. Ltd. (Milan, Italy) and were used without further purification.

2-Oxindole (**3**) is commercially available. The imidazo[2,1-*b*]thiazolylchromen-2-ones **1a**,**b** [27,28], 5-methoxyindolin-2-one **4** [29] and 1,3-dihydro-2H-benzo[g]indol-2-one **5** [30] were prepared according to the literature.

#### 4.1.1. General Procedure for the Synthesis of the Aldehydes 2

The Vilsmeier reagent was prepared at 0–5 °C by dropping POCl_3_ (54 mmol) into a stirred solution of DMF (65 mmol) in CHCl_3_ (5 mL). The appropriate starting compound **1** (15 mmol) was suspended in CHCl_3_ (20 mL). The mixture thus obtained was dropped into the Vilsmeier reagent while maintaining stirring and cooling. The reaction mixture was kept for 1 h at room temperature and under reflux for 4–6 h (according to a TLC test). Chloroform was removed under reduced pressure, the resulting oil was poured onto ice and the precipitate thus obtained was collected by filtration. The crude aldehydes were crystallized from ethanol.

*6-(2-oxo-2H-chromen-3-yl)imidazo[2,1-b]thiazole-5-carbaldehyde,***2a**, Yield 75% mp 301–302 °C IR (Nujol) ν_max:_ 1707, 1642, 1326, 1265 cm^−1^; ^1^H-NMR (DMSO-*d_6_*): δ 7.43 (1H, td, cum, *J =* 7.8, *J =* 1.1) 7.50 (1H, d, cum, *J* = 7.8) 7.63 (1H, d, th, *J* = 4.4) 7.71 (1H, td, cum, *J* = 7.8, *J* = 1.1) 7.91 (1H, dd, cum, *J* = 7.8, *J* = 1.1) 8.41 (1H d, th, *J =* 4.4) 8.62 (1H, s, cum) 9.99 (1H, s, CHO); ^13^C NMR (DMSO-*d_6_*): δ 116.123, 117.668, 118.985, 120.598, 121.037, 124.399, 124.944, 129.298, 132.864, 144.048, 148.333, 153.384, 154.535, 159.570, 179.915; Anal. Calcd for C_15_H_8_N_2_O_3_S (MW 296.30): C, 60.80; H, 2.72; N, 9.45; Found: C, 60.78; H, 2.80; N, 9.39.

*2-methyl-6-(2-oxo-2H-chromen-3-yl)imidazo[2,1-b]thiazole-5-carbaldehyde*, **2b**, Yield 81% mp 273–276 °C; IR (Nujol) ν_max_: 1717, 1657, 1611, 751 cm^−1^; ^1^H-NMR (DMSO-*d_6_*): δ 2.53 (3H, d, CH_3_, *J* = 1.4) 7.43 (1H, td, cum, *J* = 7.9, *J* = 1) 7.50 (1H, d, cum, *J* = 7.9) 7.69 (1H, td, cum, *J* = 7.8, *J* = 1.5) 7.90 (1H, dd, cum, *J* = 7.8, *J* = 1.5) 8.26 (1H d, th, *J* = 1.4) 8.59 (1H, s, cum) 9.96 (1H, s, CHO); ^13^C NMR (DMSO-*d_6_*): δ 13.56, 116.12, 117.94, 119.01, 120.60, 124.36, 124.95, 129.26, 130.26, 132.81, 143.85, 147.35, 153.35, 153.46, 159.61, 179.85; Anal. Calcd for C_16_H_10_N_2_O_3_S (MW 310.33): C, 61.93; H, 3.25; N, 9.03; Found: C, 61.95; H, 3.22; N, 9.01.

#### 4.1.2. General Procedure for the Synthesis of Compounds **6**–**10**

The appropriate indolin-2-one (10 mmol) was dissolved in methanol (100 mL) and treated with the equivalent of the appropriate imidazo[2,1-*b*]thiazole-5-carbaldehyde **2** and piperidine (1 mL to obtain compounds **6, 8, 10**) or 37% hydrochloric acid (1 mL to obtain compounds **7, 9**). The reaction mixture was refluxed for 8–13 h (according to a TLC test), and the precipitate formed on cooling was collected by filtration.

*(Z)-3-((6-(2-oxo-2H-chromen-3-yl)imidazo[2,1-b]thiazol-5-yl)methylene)indolin-2-one,***6**, Yield 63%; m.p. 301–302 °C; IR (Nujol) ν_max_: 1719, 1686, 1606, 724 cm^−1^; ^1^H-NMR (DMSO-d_6_): δ 6.87 (1H, d, ind-7, *J* = 7.8) 6.97 (1H, td, ind-5, *J* = 7.8, *J* = 1) 7.21 (1H, td, ind-6, *J* = 7.8, *J* = 1) 7.37 (1H, td, cum-6, *J* = 7.8, *J* = 1) 7.41 (1H, d, th, *J* = 4.4) 7.43 (1H, dd, cum-8, *J* = 7.8, *J* = 1) 7.62 (1H, td, cum-7, *J* = 7.8, *J* = 1) 7.67 (1H, d, ind-4, *J* = 7.8) 7.86 (1H, dd, cum-5, *J* = 7.8, *J* = 1) 7.87 (1H d, th, *J* = 4.4) 7.96 (1H, s, CH) 8.40 (1H, s, cum-4) 10.48 (1H, s, NH, ex D_2_O). ^13^C NMR (DMSO-*d_6_*): δ 109.35, 113.23, 115.87, 119.29, 119.83, 120.27, 120.86, 120.95, 122.00, 123.21, 124.45, 124.59, 124.82, 128.81, 128.86, 131.85, 140.97, 141.18, 143.36, 151.03, 153.03, 158.12, 166.62; Anal. Calcd for C_23_H_13_N_3_O_3_S (MW 411.44): C, 67.14; H, 3.18; N, 10.21; Found: C, 67.10; H, 3.22; N, 10.17.

*(Z)-5-methoxy-3-((6-(2-oxo-2H-chromen-3-yl)imidazo[2,1-b]thiazol-5-yl)methylene)indolin-2-one,***7**, Yield 63%; m.p. 308–310 °C; IR (Nujol) ν_max_: 1715, 1693, 1604, 1203 cm^−1^; ^1^H-NMR (DMSO-d_6_): δ 3.75 (3H, s, OCH_3_) 6.73 (1H, d, ind-7, *J* = 8.6) 6.81 (1H, dd, ind-6, *J* = 8.6, *J* = 2.4) 7.39 (2H, m, cum-6 + ind-4) 7.45 (2H, m, cum-8 + th) 7.64 (1H, td, cum-7, *J* = 8.1, *J* = 1.5) 7.87 (1H, dd, cum-5, *J* = 8.1, *J* = 1.5) 7.94 (1H, d, th, *J* = 4.4) 7.95 (1H, s, CH) 8.39 (1H, s, cum-4) 10.31 (1H, s, NH, ex D_2_O); ^13^C NMR (DMSO-d_6_): δ 56.61, 107.63, 110.62, 114.77, 115.72, 116.85, 120.18, 120.52, 121.91, 122.98, 123.49, 125.58, 126.18, 127.05, 129.89, 132.93, 135.91, 142.20, 143.07, 151.56, 153.98, 155.49, 159.01, 167.632; Anal. Calcd for C_24_H_15_N_3_O_4_S (MW 441.46): C, 65.30; H, 3.42; N, 9.52; Found: C, 65.31; H, 3.44; N, 9.49.

*(Z)-3-((2-methyl-6-(2-oxo-2H-chromen-3-yl)imidazo[2,1-b]thiazol-5-yl)methylene)indolin-2-one,***8**, Yield 71%; m.p. 306–308 °C; IR (Nujol) ν_max_: 1714, 1690, 1609, 722 cm^−1^; ^1^H-NMR (DMSO-d_6_): δ 2.46 (3H, d, CH_3_, *J* = 0.9) 6.83 (1H, d, ind-7, *J* = 7.5) 6.97 (1H, t, ind-5, *J* = 7.5) 7.21 (1H, t, ind-6, *J* = 7.5) 7.37 (1H, t, cum-6, *J* = 7.5) 7.42 (1H, d, cum-8, *J* = 7.5), 7.62 (1H, td, cum-7, *J* = 7.5, *J* = 1.3) 7.67 (2H, m, ind-4 + th) 7.85 (1H, dd, cum-5, *J* = 7.5, *J* = 1.3) 7.92 (1H, s, CH) 8.38 (1H, s, cum-4) 10.45 (1H, s, NH, ex D_2_O); ^13^C-NMR (DMSO-*d_6_*): δ 13.73, 109.32, 115.83, 118.64, 119.33, 119.96, 120.23, 120.85, 123.29, 124.42, 124.57, 124.85, 125.61, 128.81, 131.75, 140.77, 140.98, 142.22, 142.86, 150.00, 152.98, 158.09, 166.53, 168.42; Anal. Calcd for C_24_H_15_N_3_O_3_S (MW 425.46): C, 67.75; H, 3.55; N, 9.88; Found: C, 67.78; H, 3.57; N, 9.83.

*(Z)-5-methoxy-3-((2-methyl-6-(2-oxo-2H-chromen-3-yl)imidazo[2,1-b]thiazol-5-yl)methylene)indolin-2-one*, **9**, Yield 68%; m.p. 253–255 °C; IR (Nujol) ν_max_: 1726, 1694, 1610, 1204 cm^−1^; ^1^H-NMR (DMSO-d_6_): δ 2.47 (3H, d, CH_3_, *J* = 1.2); 3.75 (3H, s, OCH_3_) 6.73 (1H, d, ind-7, *J* = 8.3) 6.80 (1H, dd, ind-6, *J* = 8.3, *J* = 2.6) 7.38 (2H, m, cum-6 + ind-4) 7.43 (1H, d, cum-8, *J* = 8.3) 7.63 (1H, td, cum-7, *J* = 7.9, *J* = 1.4) 7.72 (1H, d, th, *J* = 1.2) 7.86 (1H, dd, cum-5, *J* = 7.9, *J* = 1.4) 7.91 (1H, s, CH) 8.37 (1H, s, cum-4) 10.27 (1H, s, NH, ex D_2_O); ^13^C NMR (DMSO-d_6_): δ 13.68, 55.63, 106.52, 109.82, 114.70, 115.84, 118.56, 119.27, 119.86, 120.82, 122.93, 124.57, 125.22, 125.79, 125.97, 128.48, 128.84, 131.80, 134.88, 140.69, 149.68, 152.94, 154.51, 158.01, 166.60; Anal. Calcd for C_25_H_17_N_3_O_4_S (MW 455.49): C, 65.92; H, 3.76; N, 9.23; Found: 65.94; H, 3.77; N, 9.20.

*(E)-3-((2-methyl-6-(2-oxo-2H-chromen-3-yl)imidazo[2,1-b]thiazol-5-yl)methylene)-1,3-dihydro-2H-benzo[g]indol-2-one,***10**, Yield 56%; m.p. 301–304 °C; IR (Nujol) ν_max_: 1730, 1692, 1607, 1204 cm^−1^; ^1^H-NMR (DMSO-d_6_): δ 2.37 (3H, d, CH_3_, *J* = 1.2) 6.64 (1H, d, ind-4, *J* = 8.2) 7.25 (1H, d, ind-5, *J* = 8.2) 7.35 (2H, m, cum-7 + cum-8) 7.45 (1H, d, th, *J* = 1.2) 7.50 (2H, m, ind-7 + ind-8) 7.58 (1H, td, cum-6, *J* = 8.2, *J* = 1.2) 7.78 (1H, dd, ind-6, *J* = 8.2, *J* = 1.2) 7.83 (1H, dd, cum-5, *J* = 8.2, *J* = 1.2) 7.88 (1H, s, CH) 8.12 (1H, d, ind-9, *J* = 8.2) 8.53 (1H, s, cum-4) 11.39 (1H, s, NH, ex D_2_O); ^13^C NMR (DMSO-*d_6_*): δ 13.66, 115.16, 115.81, 117.27, 119.05, 119.08, 120.39, 120.42, 120.51, 120.61, 121.83, 122.54, 124.66, 125.98, 127.00, 127.50, 127.62, 128.31, 128.84, 132.01, 133.58, 140.07, 140.66, 142.10, 150.15, 152.93, 158.09, 169.48; Anal. Calcd for C_28_H_17_N_3_O_3_S (MW 475.52): C, 70.72; H, 3.60; N, 8.84; Found: C, 70.74; H, 3.61; N, 8.80.

### 4.2. Biological Activity

#### 4.2.1. Cells

Erythroid progenitor cells (EPCs) were generated in vitro from peripheral blood mononuclear cells (PBMC), obtained from the leukocyte-enriched buffy coats of anonymous blood donors available for institutional research purposes from the Immunohematology and Transfusion Service, S.Orsola-Malpighi University Hospital, Bologna (authorization 0070755/1980/2014). Availability was granted under conditions complying with Italian privacy law. Neither specific ethics committee approval nor written consent from donors was required for this research project. PBMC, isolated by centrifugation in Ficoll-Paque Plus (GE Healthcare Bio-Sciences AB, Milan, Italy), were cultured in IMDM (Gibco) supplemented with 20% serum substitute BIT 9500 (StemCell Technologies, Monza, Italy), and enriched with erythropoietic growth factors as described [13]. The cells were maintained at 37 °C in 5% CO_2_ and used for infection experiments at day 9 ± 1, when permissiveness to B19V infection is maximal. UT7/EpoS1 cells were cultured in IMDM, supplemented with 10% FCS and 2 U/mL rhu erythropoietin, at 37 °C and 5% CO_2_

#### 4.2.2. Cell Viability and Proliferation Assays

The effects of tested compounds on cell viability and proliferation were monitored by a WST-8-based assay, a water-soluble salt reagent that produces a formazan dye upon reduction in response to metabolic activity (CCK8 assay, Microtech, Naples, Italy). For experiments, 5 × 10^4^ cells were seeded in 100 µL volumes in a 96-well culture microplate, and cultured in the absence, as control, or in the presence of different concentration of compounds added to medium for 48 h. Then, WST-8 reagent was added for the last 6 h for EPCs, or 2 h for UT7/EpoS1. The amounts of formazan dye were measured as absorbance (OD) values according to manufacturers’ instructions. Replicate net OD values were normalised with respect to the control samples and expressed as mean percentage values.

#### 4.2.3. Infection

A B19V viremic serum sample, identified in our laboratory in the course of institutional diagnostic service and available for research purposes according to Italian privacy law, was used as source of virus for the infection experiments. The viremic serum contained 10^12^ B19V (genotype 1) genome copies (geq)/mL, as determined by quantitative PCR analysis [31], and tested negative by routine diagnostic assays to other viruses, including HIV, HBV, HCV, HSV, VZV, EBV, CMV, HHV8, AdV, BKV. For infection, cells were incubated in PBS at a density of 10^7^ cell/mL, in the presence of the B19V viremic serum, diluted in PBS in order to obtain a multiplicity of infection (moi, expressed as geq/cell) of 10^3^ geq/cell. Following 2 h at 37 °C, the inoculum virus was washed twice in PBS and the cells were incubated at 37 °C in 5% CO_2_ in the respective growth medium, at the different concentrations of tested compounds, at an initial density of 10^6^ cells/mL.

#### 4.2.4. Nucleic Acid Purification and Analysis

Equal amounts of cell cultures, corresponding to 1.5 × 10^5^ cells, were collected as appropriate at 2 or 48 hpi and processed by using the Maxwell Viral Total Nucleic Acid kit on a Maxwell MDx platform (Promega, Milan Italy), following the manufacturer’s instructions, in order to obtain a total nucleic acid fraction in elution volumes of 150 µL. For quantitative analysis of viral DNA, an aliquot of the eluted nucleic acids, corresponding to ~500 cells, was amplified by qPCR using the primer pair R2210-R2355, located in the central exon of the B19V genome [32], and quantified using an external calibration curve, in a RotorQ system (Qiagen, Milan, Italy). As a control, a target sequence in the region of genomic DNA coding for 5.8S rRNA (rDNA) was amplified in parallel reactions. Amplification reactions were performed by using Maxima SYBR Green qPCR Master Mix (Thermo Scientific, Monza, Italy), including primers (obtained from Eurofins Genomics, Milan, Italy) at a final concentration of 0.3 µM. Quantitation of viral DNA was obtained by the absolute quantitation algorithm, converting quantification cycle (Cq) values to geq number using external calibration curves obtained from standard targets. For the rDNA target, a coefficient of variation of quantification cycle values for the different samples ≤ 5% was required, so that this parameter could be considered invariant and the normalisation by rDNA not necessary for the following quantitative determination of viral targets.

#### 4.2.5. Statistical Analysis

Experiments were carried out at least in duplicate series unless stated and, for each sample, quantitative determinations were carried out at least in duplicate values. Statistical analysis was carried out using GraphPad Prism version 5.00 for Windows (GraphPad Software, San Diego California, CA, USA). Two-way ANOVA (analysis of variance) followed by the Bonferroni test was used to compare data obtained in different experimental conditions.

## Figures and Tables

**Table 1:** New derivatives

COMP	R	R_1_	R_2_	R_3_
**3**		H	H	H
**4**		OCH_3_	H	H
**5**		H	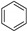	
**6**	H	H	H	H
**7**	H	OCH_3_	H	H
**8**	CH_3_	H	H	H
**9**	CH_3_	OCH_3_	H	H
**10**	CH_3_	H	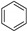	

**Table 2:** Maximal compound solubility in medium.

Compound	Concentration (μM)
**1a**	12.50
**2a**	6.25
**6**	0.78
**7**	12.50
**1b**	1.56
**2b**	0.78
**8**	3.13
**9**	12.50
**10**	6.25

**Table 3:** Inhibition of viral replication and reduction in cell viability.

Cells				Compound								
**UT7**	**2hpi**	**48hpi**	**DMSO**	**1a**	**2a**	**6**	**7**	**1b**	**2b**	**8**	**9**	**10**
Log B19V Copies ^a^	5.03	7.02	7.16	6.61	6.89	7.06	6.41	7.11	7.13	6.97	6.69	6.78
Delta Log ^b^		1.99	2.13	1.58	1.86	2.03	1.38	2.08	2.09	1.94	1.66	1.74
% viral replication ^c^		73.73	100.00	28.41	53.59	79.99	17.87	88.96	92.07	65.04	34.16	41.38
% cell viability ^c^		97.87	100.00	70.85	100.59	108.34	70.77	93.35	107.24	96.74	60.10	68.54
Ratio v/r ^d^		1.36	1.00	2.49	1.88	1.35	3.99	1.05	1.16	1.49	1.77	1.66
**EPCs**	**2hpi**	**48hpi**	**DMSO**	**1a**	**2a**	**6**	**7**	**1b**	**2b**	**8**	**9**	**10**
Log B19V Copies ^a^	5.02	7.37	7.37	7.10	7.26	7.51	7.12	7.40	7.16	6.91	7.04	7.15
Delta Log ^b^		2.35	2.35	2.08	2.23	2.49	2.09	2.38	2.13	1.89	2.01	2.13
% viral replication ^c^		100.36	100.00	54.05	76.87	137.83	55.76	108.13	60.92	34.96	46.18	60.43
% cell viability ^c^		112.41	100.00	53.92	87.09	91.14	74.43	88.10	96.20	82.78	54.43	82.53
Ratio v/r ^d^		1.12	1.00	1.00	1.13	0.66	1.34	0.82	1.58	2.39	1.18	1.37

^a^ Log B19V genome copies/104 cells; ^b^ increase respective to 2 hpi sample; ^c^ compared to 1% DMSO (48 hpi); ^d^ ratio of % cell viability to % viral replication.
